# Misincorporations of amino acids in p53 in human cells at artificially constructed termination codons in the presence of the aminoglycoside Gentamicin

**DOI:** 10.3389/fgene.2024.1407375

**Published:** 2024-11-05

**Authors:** Kamila Pawlicka, Tomas Henek, Lukas Uhrik, Lenka Hernychova, Monikaben Padariya, Jakub Faktor, Sławomir Makowiec, Borivoj Vojtesek, David Goodlett, Ted Hupp, Umesh Kalathiya

**Affiliations:** ^1^ Edinburgh Cancer Research Centre, Institute of Genetics and Molecular Medicine, University of Edinburgh, Edinburgh, United Kingdom; ^2^ Research Centre for Applied Molecular Oncology, Masaryk Memorial Cancer Institute, Brno, Czechia; ^3^ International Centre for Cancer Vaccine Science (ICCVS), University of Gdańsk, Gdańsk, Poland; ^4^ Department of Organic Chemistry, Faculty of Chemistry, Gdańsk University of Technology, Gdańsk, Poland; ^5^ Laboratory of Growth Regulators, Institute of Experimental Botany, The Czech Academy of Sciences, Olomouc, Czechia; ^6^ Biochemistry and Microbiology, University of Victoria, Victoria, BC, Canada

**Keywords:** p53, stop codon, readthrough, gentamicin, NMD

## Abstract

Readthrough of a translation termination codon is regulated by ribosomal A site recognition and insertion of near-cognate tRNAs. Small molecules exist that mediate incorporation of amino acids at the stop codon and production of full-length, often functional protein but defining the actual amino acid that is incorporated remains a challenging area. Herein, we report on the development a human cell model that can be used to determine whether rules can be developed using mass spectrometry that define the type of amino acid that is placed at a premature termination codon (PTC) during readthrough mediated by an aminoglycoside. The first PTC we analyzed contained the relatively common cancer-associated termination signal at codon 213 in the p53 gene. Despite of identifying a tryptic peptide with the incorporation of an R at codon 213 in the presence of the aminoglycoside, there were no other tryptic peptides detected across codon 213 that could be recovered; hence we constructed a more robust artificial PTC model. P53 expression plasmids were developed that incorporate a string of single synthetic TGA (opal) stop codons at S^127^P^128^A^129^ within the relatively abundant tryptic p53 peptide 121-SVTCTYSPALNK-132. The treatment of cells stably expressing the p53-TGA^129^ mutation, treated with Gentamicin, followed by immunoprecipitation and trypsinization of p53, resulted in the identification R, W, or C within the tryptic peptide at codon-TGA^129^; as expected based on the two-base pairing of the respective anticodons in the tRNA to UGA, with R being the most abundant. By contrast, incorporating the amber or ochre premature stop codons, TAA^129^ or TAG^129^ resulted in the incorporation of a Y or Q amino acid, again as expected based on the two base pairings to the anticodons, with Q being the most abundant. A reproducible non-canonical readthrough termination codon-skip event at the extreme C-terminus at codon 436 in the SBP-p53 fusion protein was detected which provided a novel assay for non-canonical readthrough at an extreme C-terminal PTC. The incorporation of amino acids at codons 127, 128, or 129 generally result in a p53 protein that is predicted to be ‘unfolded’ or inactive as defined by molecular dynamic simulations presumably because the production of mixed wild-type p53 and mutant oligomers are known to be inactive through dominant negative effects of the mutation. The data highlight the need to not only produce novel small molecules that can readthrough PTCs or C-terminal termination codons, but also the need to design methods to insert the required amino acid at the position that could result in a ‘wild-type’ functional protein.

## 1 Introduction

Translation from messenger RNA (mRNA) onto protein is a crucial step in the gene expression pathway and dysregulation of this process results in risk of disease (Tahmasebi et al., 2018). Translation starts when an initiation codon (most commonly AUG) enters the A-site of the ribosome, with a complementary base pairing of aminoacyl-tRNA anticodon, loaded with methionine, and terminates when site A of the ribosome encounters one of the stop codons UAA, UAG, or UGA (Hughes and Chevance, 2018). In humans, the only tRNA to recognize these termination codons is tRNA[Ser]Sec, which carries the amino acid selenocysteine (Labunskyy et al., 2014). After the translational machinery reaches a termination site, competition occurs between the termination complex composed of at least two eukaryotic release factors (eRFs) 1 and 3, and near-cognate tRNAs, that recognise two out of three bases in stop codon (Muramatsu et al., 2001). The eRF1 subunit entering the A site of the ribosome recognizes the termination codon and facilitates release of the mRNA from the ribosome, by factors such as ABCE1 leading to dissociation of the ribosome (Pisarev et al., 2010). Translation termination factors are recruited in more than 99.9% of cases, most likely due to a lower energetic stability of termination codon recognition by a near-cognate tRNA (Palma and Lejeune, 2021).

Sometimes the translation machinery may not recognise terminating codons and continue to extend the protein in C-terminal domain, this process is called termination codon readthrough (Lombardi et al., 2022). Readthrough can be stimulated by a dedicated regulatory environment, leading to generation of different protein isoforms (Manjunath et al., 2022). For example the readthrough-extended lactate dehydrogenase subunit LDHBx has been shown to co-import LDHA, the other LDH subunit, into peroxisomes, where it is involved in the regeneration of redox equivalents for peroxisomal β-oxidation (Schueren et al., 2014). Small molecules exist that interfere with premature termination codon (PTC) recognition and lead to production of full-length, often functional protein. Drugs that have been proven to have the highest impact on the PTC readthrough are aminoglycosides (Wangen and Green, 2020). Their efficiency to interfere with PTC recognition has been evaluated for different genes (Bidou et al., 2004; Borgatti et al., 2020). Aminoglycosides exert their PTC readthrough activity by binding at the decoding center of the eukaryotic ribosome. Binding alters the ability of translation termination factors to accurately recognize a PTC. Consequently, aminoglycosides increase the frequency of pairing of near-cognate aminoacyl-tRNAs to the PTC and enable formation of full-length protein (Baradaran-Heravi et al., 2016). There is a correlation between stop codon sequence and readthrough in response to aminoglycoside treatment where preferences show the order of UGA > UAG > UAA, as well as surrounding bases (Wangen and Green, 2020).

A nonsense mutation is a type of point mutation that occurs when a codon for an amino acid is changed to a PTC, causing the translation of the protein to halt earlier than expected (Karousis and Muhlemann, 2022). Transcripts carrying PTC form often defective truncated proteins, that could be non-functional, or also mutant gain-of-function, and have been shown to lead to many pathologies, such as cystic fibrosis, Duchenne muscular dystrophy, or cancer, the latter of which includes genes such as the tumour suppressors p53 and BRCA1 (Allamand et al., 2008; Bidou et al., 2022; Abreu et al., 2022). The efficiency of the readthrough of a PTC depends on competition between stop codon recognition by a class I release factor and decoding of the stop codon by a near-cognate tRNA (Lee et al., 2022). This decoding leads to the readthrough, by incorporating an amino acid in place of the stop codon and to the synthesis of an extended protein that ends at the next stop codon present in the same reading frame (Baradaran-Heravi et al., 2016). It has been shown, that when used in cells carrying PTCs in p53, pharmacological NMD (nonsense-mediated mRNA decay) inhibition combined sometimes with a PTC readthrough drug led to restoration of full-length p53 protein, upregulation of p53 downstream transcripts, and cell death (Martin et al., 2014; Yamashita et al., 2001; Cheng et al., 2022).

As cancers can induce novel frame-shift PTC mutations, these data highlight the utility in developing robust rules that predict and/or tune the type of mutated peptide sequences that can be induced by nonsense-suppression. Identifying the actual amino acid at a PTC generally requires the use of mass spectrometry to define the peptide sequence, which perhaps limits the routine study of this problem. Such insights might accelerate the development of strategies, for example, that can produce novel mutated neoantigens that mediate tumour rejection. A prior study using luciferase in yeast, as a model gene with a PTC, developed specific rules on the type of amino acid incorporated upon PTC readthrough after NMD-family gene deletion (such as UPF1) or the use of aminoglycosides (Roy et al., 2015).

In this report, we aimed to develop a PTC model using a specific gene (p53) in a human cell model that exploits the ability of mass spectrometry to identify the amino acids inserted when a PTC occupies the ribosomal A site. Although prior reports have shown that certain molecules can stimulate readthrough of a termination codon in p53 (Palomar-Siles et al., 2022; Floquet et al., 2011), to our knowledge there has been no direct evidence on the amino acids incorporated at such premature termination codons in p53. Methodologies we developed using a human cell model can be used to begin to manipulate or fine-tune the quantitative incorporation of specific amino acids in p53 during the readthrough process and control the type of functional protein produced after PTC readthrough and/or the C-terminal termination codon readthrough.

## 2 Results and discussion

### 2.1 Gentamicin leads to restoration of full-length p53 protein

**FIGURE 1:**
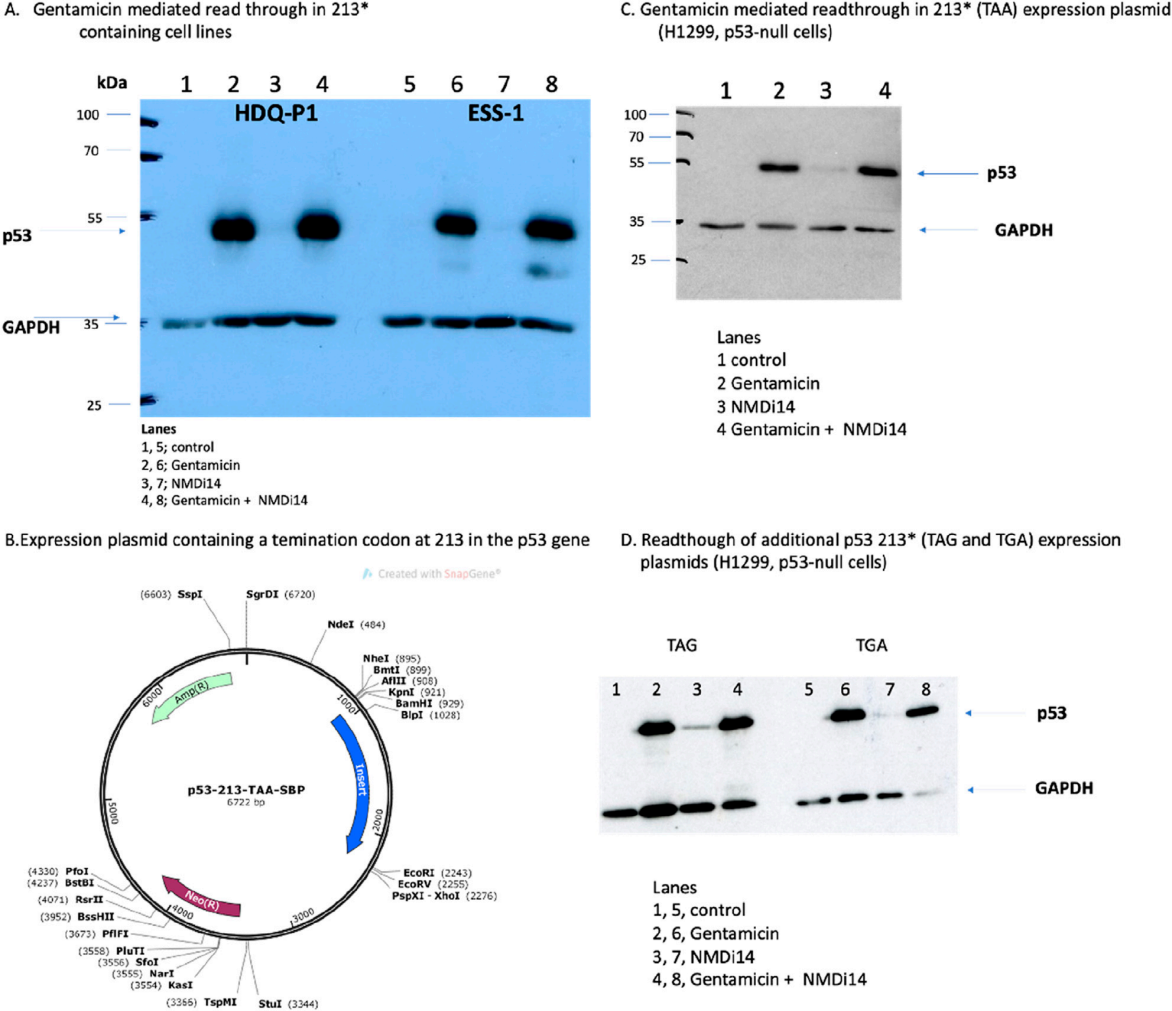
The use of a synthetic plasmid expressing nonsense mutations in the p53 gene as a model to study readthrough of a gene with a premature termination codon. **(A)** The effects of p53 protein production in the indicated cell lines (HDQ-P1 and ESS-1) after treatment with either an NMDi14 inhibitor or Gentamicin, as indicated. Lysates were immunoblotted with antibodies to either p53 protein or GAPDH as indicated. **(B, C)** The effects of p53 protein production in the H1299 cell line expressing the p53-213-STOP gene (described in B with a TAA codon) with either the NMDi14 inhibitor or Gentamicin, as indicated. Lysates were immunoblotted with antibodies to either p53 protein or GAPDH as indicated. **(D)** Additional codon 213 termination codon mutants (TAG and TGA) were transfected into the H1299 cell line and after treatment with either an NMDi14 inhibitor or Gentamicin, as indicated, the lysates were immunoblotted with antibodies to either p53 protein or GAPDH. The data shows that all three 213 termination codon mutant forms of p53 can express full-length protein when H1299 cells are treated with Gentamicin.

Some cancers such as oesophageal adenocarcinoma have a high frequency of PTC mutations (∼30%; from pan-cancer atlas libraries; cBIOPORTAL); but in particular approximately 10% of all human cancers are reported to contain p53 PTCs (Zhang et al., 2017). As such, the p53 gene is a good model to begin to address what types of amino acids can be incorporated at a PTC upon treatment with agents that can stimulate amino acid misincorporation at these codons. The ultimate therapeutic aim to “tune” the type of amino acid incorporated during PTC readthrough would be to reactivate the wild-type functions of p53. Since the most common nonsense mutation occurring in the TP53 gene is at R213X (Zhang et al., 2017), the type of the amino acid incorporated at that site (such as the wild-type Arg itself) upon drug treatment could give a therapeutic solution by the reactivation of wild-type p53 function. To assess whether NMD inhibition and/or PTC readthrough using the aminoglycoside Gentamicin leads to the restoration of p53 protein expression, ESS1 and HDQ-P1 cell lines carrying the R213X allele have been used. As previously reported (Baradaran-Heravi et al., 2021), Gentamicin can lead to full-length p53 protein production in such cell lines (Figure 1A, lanes 2 and 6 vs. lanes 1 and 5). The NMD inhibitor (NMDi14) is not as effective in the production of full-length p53 (Figure 1A, lanes 3 and 7). Combining Gentamicin with NMDi14 does not lead to detectable synergy in the production of full-length p53 protein (Figure 1A, lanes 4 and 8).

To study PTC readthrough of the three possible p53-stop codon alleles (ocher, amber, and opal), an artificial cell model was constructed in which the three different mutated p53 alleles were transfected into a cancer cell that is null for p53 (the H1299 cell line). We created and transfected a plasmid encoding the p53 gene (with the termination codon R213-TAA) fused at its C-terminus with the Streptavidin-binding protein tag (SBP) into the H1299 cell line (Figure 1B). The SBP-tag at the C-terminus was incorporated to potentially enable isolation of the full-length p53 protein from the cell lysates using streptavidin beads after the successful readthrough for subsequent trypsinization and peptide identification by mass spectrometry.

Western blot analysis revealed that treatment of cells expressing the R213-TAA gene do not produce full-length p53 protein (Figure 1C, lane 1). Immunoblotting lysates from cells transfected with the R213-TAA expression plasmid and then incubated with the NMD inhibitor (NMDi14) induced weak production of p53 protein (Figure 1C, lane 3 vs. 1). However, under the same conditions, Gentamicin induced more pronounced restoration of the full-length p53 protein (Figure 1C, lane 2 vs. 1). Combined treatment of the NMDi14 and Gentamicin did not synergize to induce more full-length p53 to appreciable levels, than Gentamicin alone (Figure 1C lane 2 vs. lane 4). Similarly, the other R213X expression plasmids with the TAG or TGA termination codons were also able to be used with Gentamicin to produce full-length p53 (Figure 1D). Together, these data indicate that the H1299-p53 null cell line can be used as a model upon transfection with a plasmid containing C-terminally tagged SBP-tagged p53 with a stop codon at the 213 position.

### 2.2 Limited recovery of a tryptic peptide at the readthrough termination codon R213X

After establishing the efficiency of the termination codon read through by western blot analysis, cell lysates were incubated with streptavidin-coated beads to pull down SBP-tagged p53 protein. The captured proteins were then trypsinized on the beads and analysed by mass spectrometry. The yield of the SBP-p53 protein pulldown was relatively low (data not shown), when using this method, which led to many rounds of unsuccessful optimization before we switched the approach and used anti-p53 antibody (DO-1)-cross-linked protein G beads. Using the DO-1 monoclonal antibody (which binds to the N-terminus of p53, before the PTC), we recovered more p53 protein (data not shown); however, on-bead trypsinization was poor (data not shown). Thus, the material captured in the DO-1 antibody bead precipitation was eluted with SDS sample buffer and we used SDS-gel electrophoresis to recover protein followed by in-gel trypsinization (Supplementary Figure S1). This method yielded better peptide recovery as evidenced by the number of tryptic peptides identified after the addition of Gentamicin.

**FIGURE 2:**
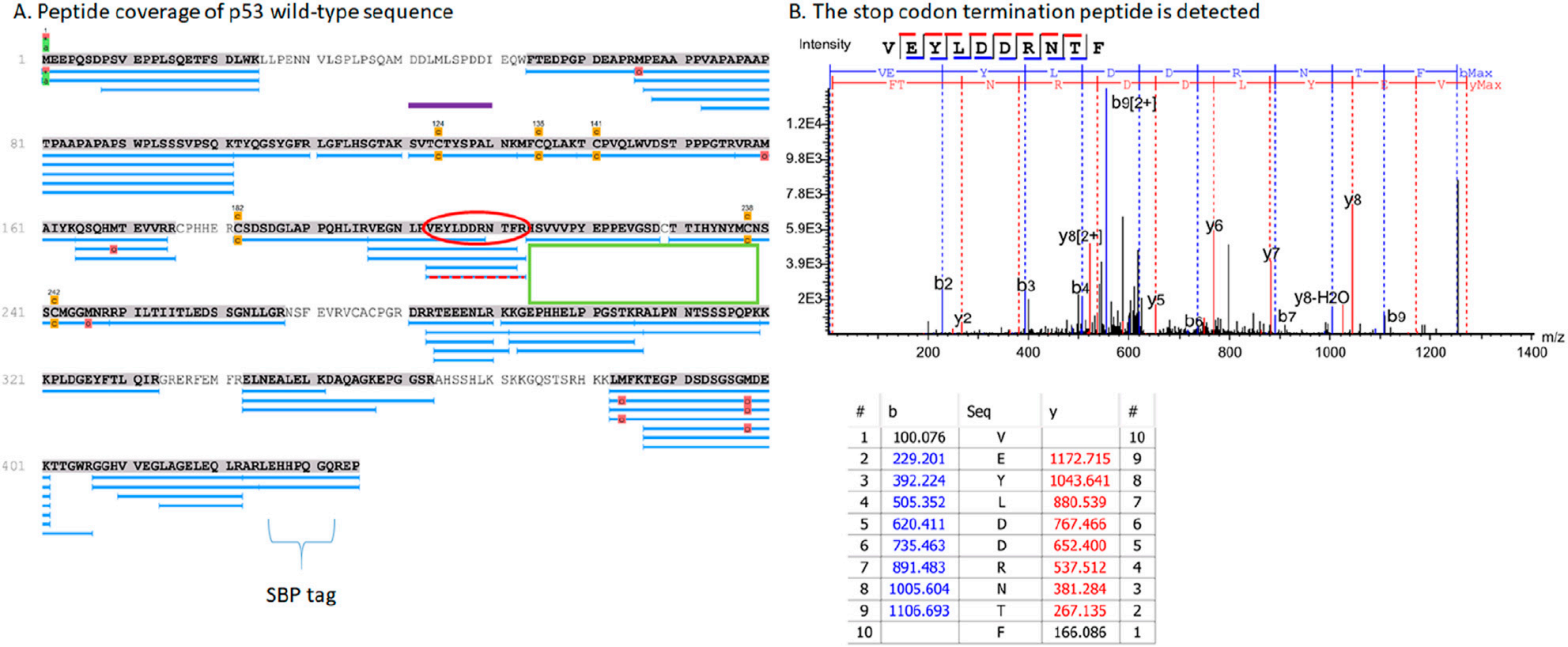
Tryptic peptide coverage of the p53 protein expressed from the R213X expression plasmid. **(A)** H1299 cells were transfected with plasmid expressing the TGA termination codon at codon 213 and treated with Gentamicin as in Figure 1. Samples were processed as indicated in the methods to produce tryptic peptides from the gel slice (Supplementary Figure S1). The figure shows representative tryptic peptide coverage of p53 as the blue annotated lines with several gaps. The 213 PTC codon peptide containing an R insertion is highlighted by a red dotted line. The low recovery and/or coverage of p53 tryptic peptide sequence C-terminal to the PTC is highlighted by the green box. The overlapping tryptic peptide coverage of p53 is staggered C-terminal to the 213 PTC position (overlapping blue lines from amino acids ∼270 through to the C-terminal SBP tag. The SBP tryptic peptide sequence (brackets) is also detected at the C-terminus (425-LEHHPQGQREP-435) which also confirms the readthrough of p53. The purple line highlights the localization of the reproducibly recovered tryptic peptide (121-SVTCTYSPALNK-132) that we use to introduce a termination codon as a model readthrough peptide (See Supplementary Figures S2–S4). **(B)** An example of a tryptic peptide that terminates at the PTC at codon 213; 203-VEYLDDRNTF*-212 Additional parameters include; scan: 23191; −10LgP PEAKS score: 50.71; mass of the precursor peak: 1270.58, m/z 636.30 (z = 2); error in ppm: 0.9; RT 33.54 min); see Figure 4B, line 5b, columns 1-3. Additional representative MS/MS spectra of tryptic peptides 203-VEYLDDRNTFR-213, which incorporates the wild-type arginine amino aid at codon 213, and 121-SVTCTYSPALNK-132 which forms our model PTC peptide, are shown in Supplementary Figures S2A, B.

**FIGURE 3:**
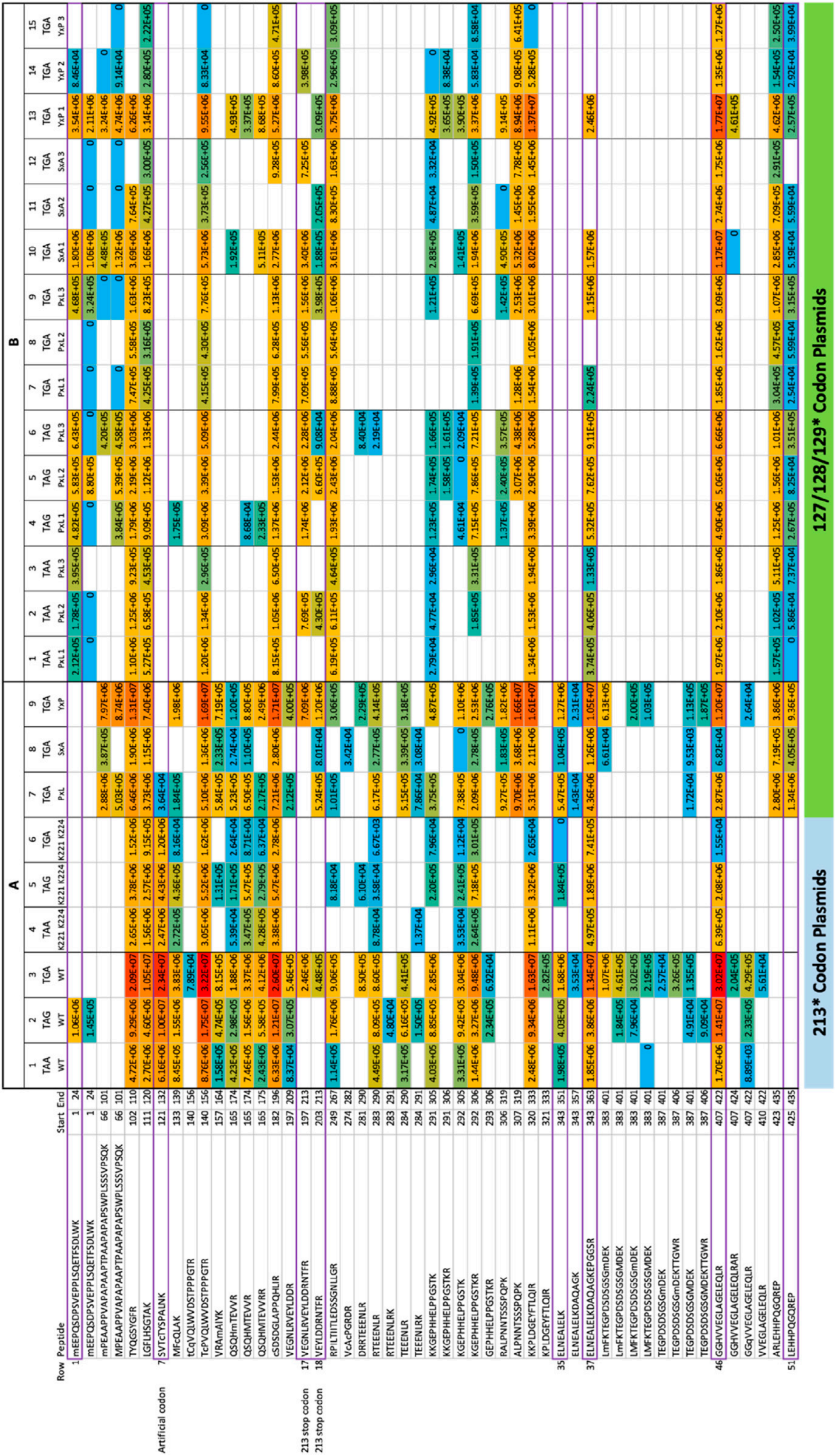
Overall summary of peptide identification that corresponds to the wild-type p53 sequence over all the replicates. **(A)** The transient transfection methodology. We initially transiently transfected three different plasmids containing the three different nonsense termination codons at codon 213 of the p53 gene (columns 1–3); peptides matching the wild-type p53 sequence using PEAKS that were acquired from the 213-TAA plasmid are presented in Column 1; the 213-TAG plasmid expression peptides are Column 2; and the 213-TGA plasmid expressing peptides are in Column 3. Overall, 51 different overlapping tryptic and semi-tryptic peptides were detected that match the wild-type p53 sequence in all three samples (see Figure 2A as a representative summary of overall peptide sequence coverage in the p53 gene and a detailed summary of specific peptides in columns 1–3, row labelled 1 at the top and row 51 at the bottom). A second independent transient transfection of the three different p53 expression plasmids was also performed (columns 4–6) containing the three different termination codons of p53 at codon 213 except these also contained two E-K mutations (221-EPPE-224; 221-KPPK-224) in order to introduce tryptic cleavage sites near the 213 termination codon in attempts to recover more tryptic peptides from this region; peptides matching the wild-type p53 sequence using PEAKS that were acquired from the 213-TAA/221-KPPK-224 plasmid are presented in Column 4; the 213-TAG/221-KPPK-224 plasmid expression peptides are Column 5; and the 213-TGA/221-KPPK-224 plasmid expressing peptides are in Column 6. A third experiment involved transient transfection of three different plasmids with the same TGA mutation at codons 127, 128, and 129 (Figure 3A, columns 7–9). **(B)** The stably integrated p53 gene *methodology*. A fourth independent experimental set was performed in which, after transfection, cells were selected with new sets of plasmids containing a hygromycin resistance gene for 4 weeks in the presence of Hygromycin B to maintain in culture only those cells that are carrying the integrated, tagged p53 with the nonsense mutations (Figure 3B). The overall wild-type p53 tryptic peptide recovery was very similar (Figures 3A vs. Figure 3B), although more peptides were recovered in the transient transfection vs. the stably integrated plasmid (Figures 3A vs. Figure 3B; rows 1–51). This might be due to the fact that transient transfection can produce an artificially high level of p53 protein, whereas stable cells which integrated the plasmid might be selected for the production of lower amounts of p53. Finally, in addition to the use of PEAKS to identify and quantify peptides, we also used Proteome Discoverer™ to quantify peptides (Supplementary Figure S5), with generally consistent results compared to PEAKS (Supplementary Figures S2–S4; Figure 2B). These data have: (i) the codon 129 (PxL) expression plasmids in triplicates (columns 1–3, TAA; columns 4–6, TAG; and columns 7–9, TGA); (ii) the codon 128 (SxA) expression plasmids in triplicate (columns 10–12, TGA); (iii) the codon 127 (YxP) expression plasmids in triplicate (columns 13–15, TGA). The numbers in each box represent ion intensity.

We initially transiently transfected three different plasmids containing the three different termination codons at 213; Figure 2A is a representative summary and detailed summary of specific peptides is in Figure 3 (columns 1–3, row labelled 1 at the top and row 51 at the bottom). Peptides matching the wild-type p53 sequence using PEAKS that were acquired from the 213-TAA plasmid are presented in Figure 3A-Column 1; the 213-TAG plasmid is in Figure 3A-Column 2; and the 213-TGA plasmid is in Figure 3A-Column 3. Overall, 51 different overlapping tryptic and semi-tryptic peptides were detected that match the wild-type p53 sequence in all three samples (Figure 3A). A second independent transient transfection of three different plasmids with 221-EPPE-224 to 221-KPPK-224 mutations was also performed to add new trypsin sites adjacent to the 213 termination codon (Figure 3A, columns 4–6). A third experiment involved transient transfection of three different plasmids with the same TGA mutation at codons 127, 128, and 129 (Figure 3A, columns 7–9, see below for the logic in using codon 127, 128, and 129 mutations). Finally, a fourth independent experiment was performed in which, after transfection, cells were selected with new sets of plasmids containing a hygromycin resistance gene for 4 weeks in the presence of Hygromycin B to maintain in culture only those cells that are carrying the integrated, tagged p53 with the nonsense mutations (Figure 3B). In general, the overall wild-type p53 tryptic peptide recovery was very similar (Figures 3A vs. Figure 3B), although more peptides were recovered in the transient transfection vs. the stably integrated plasmid (Figures 3A vs. Figure 3B; rows 1–51). This might be due to the fact that transient transfection can produce an artificially high level of p53 protein, whereas stable cells which integrated the plasmid might be selected for the production of lower amounts of p53. Finally, in addition to the use of PEAKS to identify and quantify peptides, we also used Proteome Discoverer™ to quantify peptides (Supplementary Figure S5), with generally consistent results compared to PEAKS (Supplementary Figures S2–S4; Figure 2B).

**FIGURE 4:**
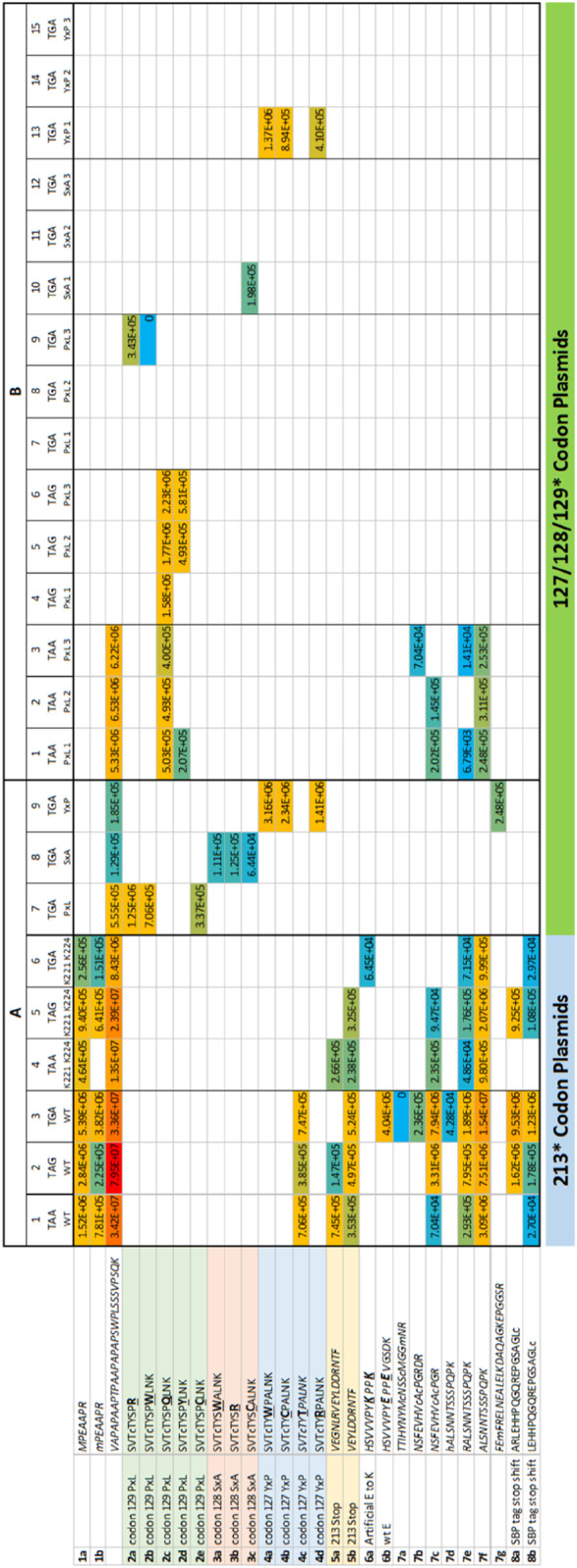
Overall summary of termination codon readthrough or variant derived peptides in all replicates. The data summarized are from the same experimental data as in Figure 3 except the data contain peptide sequences that do not match wild-type p53. **(A)** Transient transfection. The 213-TAA p53 plasmids are presented in Column 1; the 213-TAG plasmid expression peptides are Column 2; and the 213-TGA plasmid expressing peptides are in Column 3. Three different p53 expression plasmids contained the three different termination codons of p53 at position 213 except these also contained two E-K mutations (221-EPPE-224; 221-KPPK-224) in order to introduce tryptic cleavage sites near the 213 termination codon in attempts to recover more tryptic peptides from this region; the 213-TAA plasmid peptides are presented in Column 4; the 213-TAG plasmid expression peptides are Column 5; and the 213-TGA plasmid expressing peptides are in Column 6. A third experiment used three different plasmids with the same TGA mutation at codons 127, 128, and 129 (columns 7–9). **(B)**. Stably expressing p53 cells. As in Figure 8, the same samples are present; (i) the codon 129 (PxL) expression plasmids in triplicates (columns 1–3, TAA; columns 4–6, TAG; and columns 7–9, TGA); (ii) the codon 128 (SxA) expression plasmids in triplicate (columns 10–12, TGA); (iii) the codon 127 (YxP) expression plasmids in triplicate (columns 13–15, TGA). The numbers in each box represent ion intensity.

Mass spectrometric analysis first demonstrated that the semi-tryptic peptides terminating at the PTC, 197-VEGNLRVEYLDDRNTF*-212 and 203-VEYLDDRNTF*-212 can be detected (Figure 2B; and in Figure 4A; Columns 1–3 (row 5A and 5B)), after treating cells with Gentamicin, immunoprecipitation with DO-1, processing by SDS-gel electrophoresis, and trypsinization. However, peptide sequence reading through the PTC at the 213 position was often not detected reproducibly. The exception was the 213-TGA plasmid (in Figure 3A-Column 3; lines 17 and 18) which detected the peptide 197-VEGNLRVEYLDDRNTFR-213 and 203-VEYLDDRNTFR-213, respectively (intensity=2.46E+6 and 4.48E+5, AU (arbitrary units), respectively)). Both tryptic peptides (197-VEGNLRVEYLDDRNTFR-213 and 203-VEYLDDRNTFR-213) results from an Arg incorporation at the 213-termination codon and which also simultaneously produces a tryptic cleavage site (Arg213; Supplementary Figure S2A). The latter data are consistent with the concept that non-canonical base pairings between the stop codon UGA mRNA can be stabilised by Gentamicin to accommodate the tRNA-Arg containing the anti-codon 5′-UCG-3′; where the 3′-G of the anti-codon forms hydrogen bonds with the 5′-U of the codon. In addition, in this same sample, we can also detect the C-terminal tryptic peptide resulting from Arg213 cleavage; 214-HSVVVPYEPPEVGSDK-229 (Figure 4A, column 3, row 6b).

Although we could not detect readthrough of the termination site at codon 213 using the other two 213-PTC containing plasmids (Figure 3A, columns 1 and 2, lines 17 and 18); all three plasmids could be used to detect the tryptic peptide 203-VEYLDDRNTF*-212 resulting from trypsin cleavage at Arg202 and includes Phenylalanine prior to the termination event (Figure 4A, columns 1–3, row 5b). Despite the fact that these other two 213-PTC containing plasmids (213-TAA and 213-TAG) did not yield any peptides containing a 213-codon replacement (Figure 3A, columns 1 and 2, lines 17 and 18), there were several peptides detected C-terminal to the codon 213 PTC using these two plasmids (213-TAA and 213-TAG) indicating that readthrough event had occurred in these samples. One example includes the peptide from the tetramerization domain of p53: with the core peptide 343-ELNEALELK-351 (Figure 3A, columns 1 and 2, line 35). Another example includes an amino acid sequence in the extreme C-terminal SBP tag; 407-GGHVVEGLAGELEQLR-422 (Figure 3A, columns 1–3, line 46).

Apart from Arg residues, we could not detect other possible amino acids incorporated at codon 213, possibly because the resulting tryptic peptide was too long with the next available trypsin cleave site at codon 213 and possible yield recovery of this larger tryptic peptide is poor. That is, it was serendipitous that the R incorporation at codon 213 using the 213-TGA containing plasmid produced an amino acid that can be cleaved by trypsin to produce a relatively stable and small tryptic peptide.

As such we created three new 213-PTC encoding plasmids in which the sequence 221-EPPE-224 was replaced with two lysine residues to generate 221-KPPK-224 along with all three PTCs (Figures 3A, 4A, columns 4–6). We reasoned that this might allow for capture of smaller tryptic peptides. All three 221-KPPK-224 encoding plasmids with termination codons at 213 including TAA, TAG, and TGA, could all be used to produce full-length p53 protein upon Gentamicin addition (data not shown). These data suggest that the 221-EPPE-224 and 221-KPPK-224 encoding p53 plasmids act equivalently.

These 221-KPPK-224 encoding variant plasmids were similarly transiently transfected into H1299 cells and processed for immunoprecipitation of p53 after the addition of Gentamicin (Figure 3A, columns 4–6). These three plasmids generated peptides that generally overlapped with the wt-p53 sequence (Figure 3A, columns 1–3 vs. columns 4–6). For example, all six plasmids shared the p53 tetramerization sequence 343-ELNEALELKDAQAGKEPGGSR-263 (Figure 3A columns 1–6, row 37) which is C-terminal to the termination codon and confirms readthrough had occurred. However, these new plasmids did not allow recovery of the peptides 197-VEGNLRVEYLDDRNTFR-213 or 203-VEYLDDRNTFR-213, Figure 3A-Columns 4–6 (lines 17 and 18). In one case, the novel tryptic peptide incorporating 221-KPPK-224 was detected (214-HSVVVPYKPPK-224), although with a very low signal intensity of 6.45E+4 (Figure 4A; column 6, row 6a). Nevertheless, these three additional plasmids provided another set of biological samples to explore what p53 derived peptides are reproducibly recovered, or not, using all six different plasmids (Figures 3A, 4A, columns 1–6).

### 2.3 Creating a stop-codon readthrough model based on the presence of an existing stable tryptic peptide derived from p53

Apart from the variable R incorporation at codon 213 in the presence of Gentamicin (Supplementary Figure S2A), the poor tryptic peptide sequence coverage around the 213-position led to exploration of other PTC positions that could be developed to allow the detection of the amino acid inserted during the readthrough event. Since the tryptic peptide 121-SVTCTYSPALNK-132 was recovered reproducibly (see Figure 3A, columns 1–6, row 7; Supplementary Figure S2B), we decided to incorporate nested termination codons within this reproducibly recovered tryptic peptide since it is stably detected in the mass spectrometer. p53 expression plasmids were developed that incorporate a string of single synthetic TGA stop codons in plasmids at S^127^P^128^A^129^ within the tryptic p53 peptide 121-SVTCTYSPALNK-132 (Figures 3A, 4A, columns 7–9). By using these plasmids, we first tested the influence of the stop codon position (127, 128, and 129), with a fixed codon (TGA), on the amino acids incorporated during PTC readthrough. The treatment of cells expressing the p53-TGA^129^ mutation, treated with Gentamicin, followed by immunoprecipitation and trypsinization of p53, resulted in the reproducible incorporation of R, W, and C in the tryptic peptide at codon-TGA^129^ (Supplementary Figure S3A–C). Quantitative analysis using PEAKS revealed that R was the most dominating substitution introduced at the codon-TGA^129^ (54.5%; intensity=1.25E+06 AU, Figure 4A, column 7, row 2a) followed by W (30.8%; intensity=7.06E+05 AU, Figure 4A, column 7, row 2b and then by C (14.5% intensity=3.37E+05 AU; Figure 4A, column 7, row 2e).

We also evaluated the p53-TGA^127^ (Ser stop) and p53-TGA^128^ (Pro stop) (Supplementary Figure S4; Figure 3A; Figure 4A, columns 8 and 9). In one independent experiment (representative example of the spectra is in Supplementary Figures S4D and S4F), W and R were equivalent at the p53-TGA^128^ codon (Figure 4A, column 8, rows 3a and 3b; intensity=1.11E+05 and 1.25E+05 AU) with the amino acid C levels lower (Figure 4A, column 8, row 3c; intensity=6.44E+04 AU). In an independent experiment using cells stably expressing p53 (Figure 4B), rather then the transient transfections (Figure 4A), we found Cysteine alone dominating at the p53-TGA^128^ (Pro stop) in one independent experiment (Figure 4B, column 10; row 3c; intensity=1.98E+5 AU). We think the variability between independent experiments done on different dates stems from the multiple steps in the pipeline; (i) variability in the covalent conjugation of the DO-1 antibody to the beads can vary the amount of p53 recovered in the immunoprecipitation, and (ii) variability in trypsinization from the gel slice can also contribute to difference in p53 peptides recovered (tryptic peptide data available on request).

**FIGURE 5:**
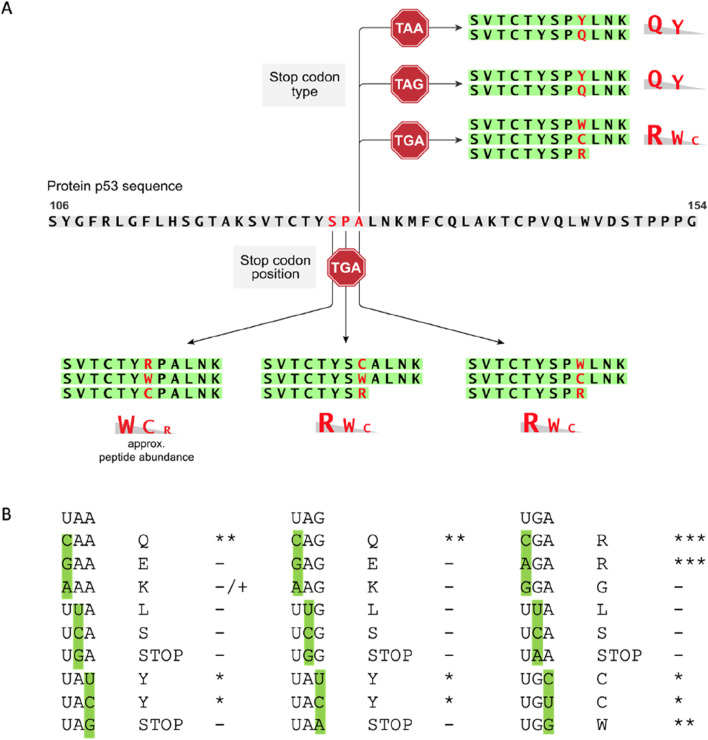
Summary of amino acids most reproducibly incorporated as a function of mRNA codon position or stop codon sequence. **(A)** The gradient of amino acid percentage incorporation is taken from data in Supplementary Figure S5 in which the amino acid incorporated is quantified using Proteome Discoverer™ version 2.2; the data also reflects the quantitation used with PEAKS (in Figures 3, 4). **(B)** We can speculate that dominating amino acids-Q at UAA; Q at UAG; R and UGA-are due to the stability of the 3’ anticodon G in each tRNA pairing with 5’ codon U in the mRNA. For example, the codons CAA (encoding Q), CAG (encoding Q), and CGA (encoding R) have anti-codons 3’-GUU-5′ (for CAA), 3’-GUC-5’ (for CAG), and 3’-GCU-5’ (for CGA), that permit a position 3-G in the tRNA anti-codon to base pair with the position 1-U in the mRNA codon. This is consistent with the fact that we observed no E incorporation at the UAA codon or UAG codons. This would have exploited the GAA codon (for E) or the GAG codon (for E) to form base pairs with the anti-codons 3’-CUU-5’ (for GAA) and 3’-CUC-5’ (for GAG), that would require a position 3-C in the tRNA anti-codon to base pair with the position 1-U in the codon mRNA. Presumably, such pyrimidine-pyrimidine base pairings are not as stable as the purine-pyrimidine pair.

In the case of the p53-TGA^127^ codon, in one independent experiment (representative spectra is in Supplementary Figure S4), W dominated at approximately 50% (Figure 4A, column 9, row 4a; intensity=3.16E+06 AU) with approximately equivalent levels of C and R detected (Figure 4A, column 9, rows 4b and 4d; intensity=2.34E+06 and 1.41E+06 AU). Thus, it appears that in two codons at positions, TGA^128^ and TGA^129^, generally accepted an R as the dominant amino acid (Figure 5A). Codon TGA^127^ however accepted W at the dominant amino acid (Figure 5A). A prior report using yeast and luciferase as the protein model with a TGA codon identified “W” as the predominant amino acid over R (Roy et al., 2015). In addition, a recent report using bacteria cells as a model identified the W as a dominant amino acid replacement at the TGA codon using mCherry-TGA-YFP reporters or the RpsG nonsense mutation gene as encoding model proteins (Zhang et al., 2020). There could be several reasons for why the R > W dominates in two out of three TGA positions in our model (Figure 5A; Figure 4A, Columns 7 and 8 vs. column 9), whereas W > R dominates in the other models. The dominance of R over W at the TGA codon might be because of the more stable G-U base pair between the position 1 (U) of the mRNA stop codon (UGA) and the position 3 (G) in the anticodon of the tRNA for Arginine, which is 3′-GCU-5′ (Figure 5B). It might also reflect differences in the cells used, levels of tRNA synthetase pathway components, as our model cell is a cancer cell with an altered genome (H1299) and the prior models used “normal” wild-type yeast cells as the model (Roy et al., 2015) or “normal” wild-type bacteria (Zhang et al., 2020). In addition, the surrounding RNA sequence can also impact on the termination codon readthrough (Dabrowski et al., 2014), which we have not evaluated in this study, and this might impact on the W > R incorporation at TGA^127^.

Various studies show that there is a difference in efficiency of the read through, depending on the PTC sequence. Next, we compared plasmids p53-STOP^129^ that encoded three different termination codons TAA, TAG, TGA (Figures 3B, 4B, columns 1–9; Supplementary Figure S3D–G; Figure 5) to test whether the preference of amino acid incorporation shifts with the stop codon change. Analysis revealed that TAA and TAG stop codons (Supplementary Figure S3D–G) demonstrated a preference towards Q insertion and less frequently Y (Figure 4B). At the TAA codon, in technical triplicates, Q was detected in the majority of samples (Figure 4B, columns 1–3, row 2c vs. 2d). Similarly, at the TAG codon, in technical triplicates, Q was detected at the highest intensity (Figure 4B, columns 4–6, row 2c vs. 2d). A prior report using yeast as the model also generally detected Q as dominating over Y at the TAG termination codon, with Q and Y incorporated at relatively similar levels at the TAA stop (Roy et al., 2015). The domination of Q over Y in our model (Figure 5A) for example in (Figure 4B, column 6, row 2c vs. 2d, compare intensity=2.23E+06 vs. 5.81E+05 AU) might be explained by the more stable G-U base pair between the position 1 (U) of the stop codon in the mRNA (UAA) and the position 3 (G) in the anticodon of the tRNA for Glutamine, which is 3’-GUU-5’ (Figure 5B). For example, presumably E is not detected as a readthrough amino acid replacement in our data because the anti-codon for E is 3’-CUU-5’ and the C-U bonding is not as stable.

### 2.4 Novel frameshift readthrough peptides from the synthetic p53 gene

**FIGURE 6:**
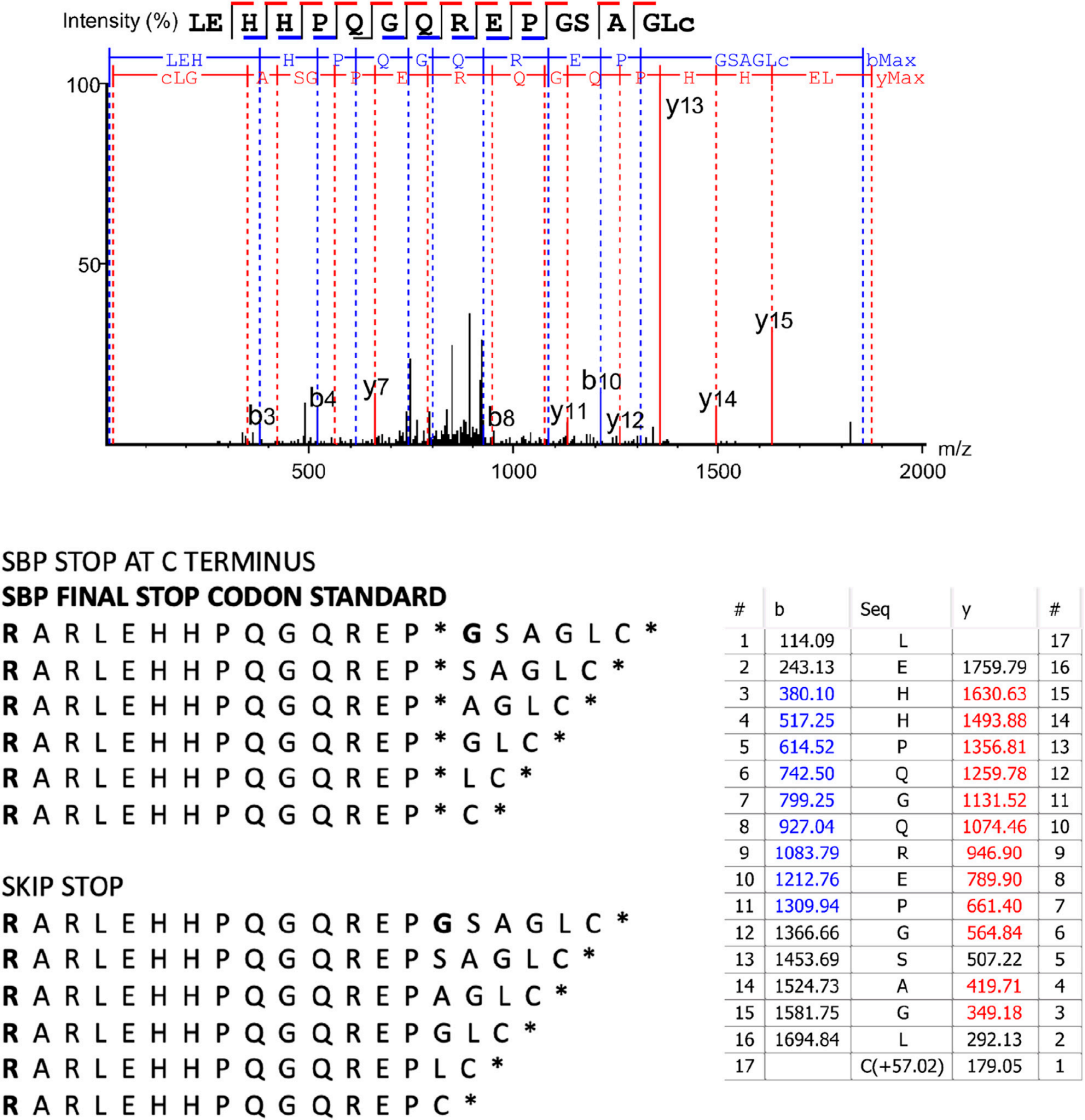
A novel termination readthrough peptide observed at the C-terminus of the SBP-p53 fusion protein. The MS/MS spectrum is representative from Figure 4A (row 8b, columns 1, 2, 3, 5, and 6). The table shows ion matches (red are y-ions and blue are b-ions). Further parameters from the measurement and evaluation are: scan: 7,129; −10lgP PEAKS peptide score: 49.86; mass of the precursor peak: 1871.87, m/z 936.94 (z = 2); error in ppm: 0.9; RT 17.38 min. The sequence of the C-terminus is shown with “SBP STOP AT C TERMINUS” that would form the sequence if an amino acid was placed at the termnination codon. The sequence of the C-terminus is also shown with the “SKIP STOP” reference sequence database where we can observed the indicated peptide sequence resulting in the termination codon skipped during translation and then in-frame translation to the next termination codon ending with the amino acid Cysteine.

Using another termination readthrough model with genetically recoded bacteria identified Q and Y at the TAG codon, but also D, V, and A amino acid, plus also new downstream peptide sequences resulting from translation frameshifting by 1, 2, 4, 7, 8, 10 or 19 bases (Ma et al., 2018). These data suggest novel protein coding space can occur with an mRNA species when aminoglycosides are used to impact on the quality of translation. In several biological replicates we can indeed observe readthrough of the most C-terminal termination codon after the SBP tag that is the result of skipping the termination codon but keeping the sequence in frame resulting in a termination event after six amino acids are incorporated (Figure 4A, row 8a and 8b, columns 1–6; Figure 6). As columns 1–6 represent 6 different plasmids (three different termination codons, and with either 221-EPPE-224 or 221-KPPK-224 sequences), transfected into different plates of cells, these data suggest that the production of this C-terminal termination codon skip-readthrough is a highly reproducible event, occurring in five out of six different biological replicates using transient transfection. However, when we made stable cells where the p53 plasmid DNA is integrated into the genome (Figures 3B, 4B) there were no detectable readthrough peptides as seen in Figure 6. As the plasmids that derived Figures 3B, 4B used stably integrated p53 plasmid, rather then transiently transfected plasmids (Figures 3A, 4A), this difference might account for the variability and points to possible mechanisms of how this non-canonical translation-skip readthrough pathway can be stimulated, modified, and studied in the future.

### 2.5 Predictions on p53 reactivation at codon 213 or codons 127–129 readthrough

The R incorporation at codon 213 in the presence of amino glycoside (Supplementary Figure S2A) restores the wild-type sequence to p53 suggesting that the TGA stop codon, at least at codon 213, can be targeted to restore wild-type p53 function. However, we could not detect p53 activity in such cells treated with Gentamicin (data not shown), suggesting that other amino acids might be incorporated at the 213 codon that create a mutant p53. In addition, as mutant p53 can inactivate wild-type p53 through mixed oligomers (Milner and Medcalf, 1991), Gentamicin might not reactivate full-length wild-type p53 function in these conditions where mutant p53 molecules are also being synthesized. Nonetheless, other studies have reported reactivation of p53 function using a gene with a R213X mutation (Zhang et al., 2017; Floquet et al., 2011) but the type of amino acids incorporated at the PTC position were undefined in these data.

**FIGURE 7:**
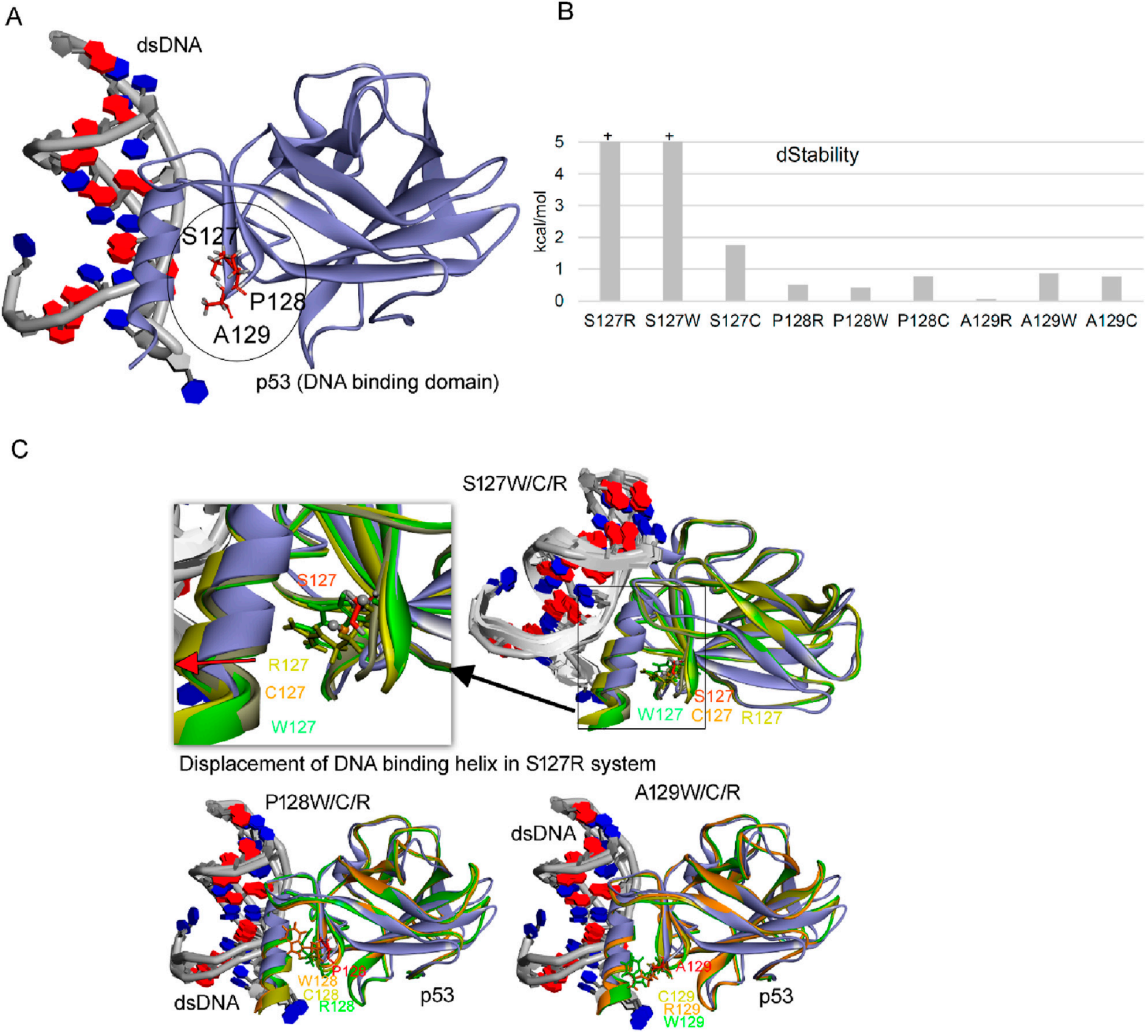
Effect of point mutation over the stability of p53 gene. **(A)** Structure of p53 with dsDNA (pdb id.: 2AC0), with stop codon readthrough derived mutations marked (Ma et al., 2018). **(B)** Change in the structural stability over the p53 protein upon inserting point mutations. **(C)** Conformational dynamics of p53 DNA binding domain (DBD) upon inserting point mutations (S127, P128, A129 with W/C/R mutations) derived from stop codon readthrough. Changes in the dStability (ΔStability) upon inserting mutations were traced using the residue scan pipeline implemented in the Molecular Operating Environment (MOE; Chemical Computing Group Inc., Montreal, QC, Canada) package. BIOVIA Discovery Studio visualizer (Dassault Systèmes, BIOVIA Corp., San Diego, CA, USA) package was used to visualise protein structures.

As these data in our model result in predictable incorporation of specific amino acids at the indicated stop codons in p53 from 127–129 (i.e., W, C, Q, Y, R), that follow the wobble rules, we wanted to estimate whether these p53 protein molecules would be active. The data demonstrate that only an Arg incorporation at codon 129 would give rise to a protein with the thermodynamic stability similar to wild-type p53, whilst all other incorporations do not yield ‘active’ p53 (Figure 7). These data suggest that to fully exploit p53 stop codon readthrough as a therapeutic option, then strategies need to be developed that by-pass the ‘default’ incorporation of W, C, Q, and Y and can produce precise amino acids at the specific codon in question that produces the wild-type p53 protein with no mutant sequences.

## 3 Conclusion

We report here on the development of a novel human cell line model using nonsense mutations in p53 as a tool to begin to define the type of amino acids incorporated during readthrough, as a function of some perturbation. Prior to this report, all studies on measuring p53 termination codon readthrough used aminoglycosides that resulted in a p53 readthrough event and a correlative increase in p53 function, did not identify what amino acid might have been incorporated at the termination codon during the readthrough event (Martin et al., 2014; Yamashita et al., 2001; Cheng et al., 2022; Palomar-Siles et al., 2022; Floquet et al., 2011) (Anastassiadis and Kohrer, 2023). By contrast to these former studies, we have applied mass spectrometry and have defined the precise amino acids that can be incorporated at four different p53 codon positions (127, 128, 129, and 213) and at one fixed codon (129) using three different nonsense codons. These amino acids that are incorporated abide by the “wobble rule” Muramatsu et al., 2001) whereby imperfect anti-codon tRNA base pairings to the codon are accepted or tolerated when a 3’ anti-codon G in a tRNA is paired with the position 5’-U in the mRNA codon (Figure 5). These data suggest that the pyrimidine-pyrimidine base pairings are not as tolerated for the readthrough event as the purine-pyrimidine pair.

Another difference between our studies and the aforementioned is that we do not see robust p53 “activation”, as a result of producing full-length p53, in response to treatment of p53 mutant cells with aminoglycosides (Figure 7). Such differences between our studies and the prior studies might be explained by the amount of Gentamicin used, the time course of the experiment, and other conditions such as type of functional assay (reporter or endogenous gene) used or the cell lines used. In addition, because our cell model predicts that even if an “activating” or wild-type amino acid is produced during readthrough of the PTC-containing p53 gene, because more than one amino acid can be incorporated at a termination codon (Figures 3–5), this can create a mixed wild-type p53/mutant p53 oligomer which is known to create a dominant negative inactive p53 oligomer (Milner and Medcalf, 1991). Thus, we would not necessarily expect that the “correct” ‘activating amino acid to be incorporated at any p53 PTC by aminoglycosides, because the readthrough event is driven by the default toleration of the 3’ anticodon G:5’ codon U base pairing. Although mixed amino acid incorporations into p53 oligomers might create inactive p53 molecules in human cancers (Milner and Medcalf, 1991), there are other clinical syndromes such as skin diseases with a PTC driver gene (Li et al., 2023) where a PTC readthrough in response to medicinal treatment produces a “full-length” protein with activity. Such events might reactivate the wild-type functions of such proteins emanating from the C-terminal portion of the protein without the negative conformational effects observed with a homo-oligomeric protein such as p53. This suggests on the surface that clinical impact of aminoglycoside medicines might be positive in some diseases with non-allosteric or non-oligomeric proteins and that in these cases we would not necessarily need to know which amino acid was incorporated at a PTC.

On the subject of mass spectrometry data, our model also mimics that observed with other bacterial and yeast based models in the identification of R, W, or C as the dominant amino acids at a TGA termination codon, as well as Q or Y at the TAA and TAG termination codons. However, there were some quantitative differences we observed (generally a R over W at TGA and Q over Y at the other two) that suggest position 1 of the codon and position 3 of the anticodon play a more important role in readthrough in the presence of Gentamicin (Figure 5). In addition, our model shows variability in the methods used to enrich for p53, which might be due to several steps including variability in the amount of DO-1 antibody bound to the Protein G beads as a batch-to-batch variation, or in the amount of p53 produced during the readthrough process, or recovery of the material from the SDS-gel using in-gel trypsinization (Supplementary Figure S1). Because of this variability, technical triplicates were required in any given experiment to allow for semi-quantitation.

The main difficulty in identifying the amino acid incorporated at any nonsense codon is that the readthrough peptide product needs to be stable using mass spectrometry with sufficient concentration. One cannot guarantee a particular region of any protein can produce a stable tryptic peptide. Indeed, in our p53 map, we observed less than 50% coverage of the entire protein over several runs even after p53 protein concentration onto Protein G beads from cell lysates using immunoprecipitation (Figure 2A; Supplementary Figure S1). This fact reduces the PTC regions we could use to study readthrough. As such we focused on a region in the N-terminus of p53 protein with wild-type amino acid sequences that produces a relatively reproducible tryptic peptide that allowed us to analyse the effects of codon change in the context of a TGA nonsense codon (127, 128, or 129) and the effects of all three nonsense codons change at one fixed position (codon 129).

An interesting observation was made recently that frameshift mutations can accompany readthrough (Ma et al., 2018), that other non-canonical amino acids (not W, R, C, Q, or A) can be observed in such context, and also that the true termination codon as well as the PTC can incorporate novel amino acids at the readthrough event. Our data on p53 also support this concept in mammalian cells; we can observe one highly reproducible novel readthrough/skip translation event using five different p53 PTC-encoding plasmids (Figure 6; Figure 4A, columns 1–6, row 8). The reproducibility of this non-canonical readthrough event provides a cell-based model to begin to identify genes involved in this non-canonical readthrough process. This also raises the question of the degree of off-target effects or toxicity of aminoglycoside-based medicines if they generally also cause translation skipping or frameshifts at the C-terminus of a given gene. Defining the depth of this novel amino acid sequence information would require methodologies to increase the yield of such non-canonical peptides by computational mass spectrometry. The non-canonical readthrough pathway also raises the interesting possibility, on the other hand, that aminoglycoside type medicines could be used to augment the production of non-canonical translational skips and frameshift neoantigens in cancer cells (Litchfield et al., 2020).

Are there ways to increase the sequence coverage of p53 protein shown in Figure 2A? We think the main difficulty in identifying readthrough peptides using mass spectrometry would be the availability, stability, or ionizability of any given tryptic “readthrough” peptide derived from p53, as is seen from gaps in certain peptides that are never recovered after trypsin treatment (Figure 2A). We do know that the transient transfection of the p53 gene, rather than cells stably expressing p53, gave rise to substantially more tryptic peptides (Figures 3A vs. 3B). This is because the transient transfection of cells can introduce higher plasmid DNA concentrations and produce more p53 protein than cells which have stably integrated a few or even one copy of the p53 gene. This means that stably expressing p53-PTC constructs in cells might be less useful than the transient transfection option. However, there may be ways to augment the tryptic peptide signals required using stably integrated reporter genes, because there are other biological factors known that can regulate termination codon readthrough. Perturbation of such genes might increase total reporter protein or, in our cell model, p53 protein levels. For example, MG132 is known to stabilize p53 protein (Maki et al., 1996) and this might increase the total levels of p53 protein and possibly more tryptic peptides. It might be also possible to elevate the signal of any given tryptic peptide by reducing the concentration of eRF3/eRF1, which in previous studies was shown to promote the production of endogenous (i.e., not transfected) full-length p53 protein with a termination codon (Baradaran-Heravi et al., 2021) or reporter proteins such as GFP (Schmied et al., 2014). In addition, the tRNA system that controls selenocysteine uses the UGA termination codon in mRNA and this might also compete with readthrough events (Vindry et al., 2023). Thus, reducing the levels of selenocysteine (Sec) tRNA (tRNA^[Ser]Sec^) might increase total p53 protein levels. However, it should be pointed out that total p53 protein levels is not an issue *per se* in these mass spectrometric methodologies because sufficient p53 protein is recovered by immunoprecipitation from several tissue culture plates after transient transfection of the p53 gene (Figures 3A vs. 3B). That is, increasing the levels of p53 protein does not necessarily increase the distribution of tryptic peptides (Figure 2A), but only the intensity of ionizable and/or recoverable tryptic peptides (Figure 3A vs. 3B). Therefore, we think that a key issue in future for detecting more readthrough events-broadening the range of peptides identified by mass spectrometry-is to use chemical cleavage methodologies that recover non-trypsin peptides or enzymes other than trypsin to try to recover more peptides that increase chances of seeing readthrough events using mass spectrometry.

One of the therapeutic outcomes of overcoming a nonsense codon is to restore the function of the normal protein in several diseases. Promoting readthrough may not always lead to therapeutic outcomes because current chemicals such as aminoglycosides rely on the default, “semi-random” misincorporation of amino acids (Figure 5) that cannot guarantee to incorporate a functional amino acid. As such, the use of precision tools to incorporate a specific amino acid at a termination codon would be required. In the case of p53, it is sensitive to inactivation at many of its codons (Milner and Medcalf, 1991). If we cannot necessarily rely on the random or default incorporation of certain amino acids at a termination codon, to reactivate p53 function-W, R, C, Y, or Q, then we would need strategies that can make amino acid choice at any one codon tuneable, such as the use of tRNA medicines that can adapt a specific amino acid upon the reading of a termination codon (Anastassiadis and Kohrer, 2023). We have tested this concept using the tRNA synthetase/tRNA cognate pair from *Methanosarcina mazei* (data not shown, manuscript in preparation) by co-transfecting p53-termination codon containing plasmids with tRNAsynthetase/tRNA pair that can incorporate a non-canonical lysine amino acid at a termination codon (Schmied et al., 2014). The co-transfection of the p53-TGA^129^ plasmid with the *M. mazei* tRNAsynthetase/tRNA_UGA_ pair can result in the production of full-length p53 which is dependent up on the addition of a specific non-canonical lysine amino acid to the media (data not shown). Such approaches can allow for selected amino acid incorporation that by-pass the ‘default’ use of tRNA carriers with an imperfect match to the termination codon. We hope the cell based p53 model we describe in this report will help accelerate such tuneable strategies to modify the PTC readthrough language for creating site-specific and accurate amino acid incorporations and that such biological events can be cross validated using the mass spectrometry methodologies we describe in this report.

## 4 Materials and methods

### 4.1 Cell culture and transfection

A375 and HDQ-P1 cell lines were grown in DMEM media (Gibco), and H1299 and ESS1 required RPMI (Gibco). Both media were supplemented with 10% (v/v) foetal bovine serum (FBS) (Labtech) and 1% (v/v) 0.5 U/mL penicillin/500 ng/mL streptomycin (P/S) (Invitrogen). Cell culture was maintained in the incubator at 37°C with 5% CO2. Once confluent, cells were sub-cultured using Trypsin–EDTA 0.5% (Gibco, Life Technologies). Plasmid DNAs were transfected using Attractene (Qiagen) according to manufacturer’s protocol on cells at 80% confluency. Transiently transfected cells were selected by culturing in the presence of hygromycin B (Gibco) for 4 weeks.

Cells were plated in 6-well plate in the density of 2*105 cells/well, after 24 h either left untreated or treated with 500 μg/mL G418 (Gibco, Life Technologies), 10 μM NMDi14 (Sigma-Aldrich) or both. The treatment was repeated 24 h later. Cells were collected the next day by washing with ice-cold PBS and scrapping the well surface. After centrifugation, cell pellets were flash-frozen on dry-ice and kept in −80°C until further processing.

#### 4.1.1 Western blot and immunoprecipitation

Cell pellets were lysed with a buffer containing 50 mM Tris-HCl, 150 mM NaCl, 1% Triton X-100 and, 5 mM EDTA and subjected to the western blot analysis of the p53 protein expression. From each cell lysate, 600 μg of protein was taken to perform immunoprecipitation assay to isolate p53 protein tagged with SBP. Lysates were incubated with DO1-conjugated protein G beads overnight in 4°C with rotation. After a series of washes, beads have been flash-frozen on dry-ice.

### 4.2 Mass spectrometry sample preparation and LC-MS/MS analysis

The elution of proteins from beads was performed by adding 1x NuPAGE LDS buffer and boiling samples for 5 min. SDS-PAGE run lasted 10 min at 120 V and the gels were stained by Coomassie Brilliant Blue G-250. Gel bands were excised out of the gel, washed with deionized water, cut into small pieces and decolored with a freshly prepared 200 mM solution of ammonium hydrogen carbonate (NH4HCO3, pH 7.8) in 40% aqueous acetonitrile (ACN) (v/v) for 20 min at 30°C and equilibrated in 50 mM NH4HCO3 (pH 7.8) in 5% aqueous ACN (v/v) for 30 min at 30°C. The supernatant was removed, and the gels were dehydrated with ACN. The supernatant was removed, and the samples were reduced by the addition of 10 mM DTT for 1 h at 60°C, followed by alkylation with 55 mM iodoacetamide in the dark for 45 min at room temperature. The supernatant was removed, and the gel pieces were washed three times with an equilibration buffer and dehydrated with acetonitrile. Trypsin digestion was carried out at 37°C overnight using Promega sequencing-grade trypsin. Digested peptides were extracted using ACN, vacuum dried and desalted using C18 micro spin columns (Harvard Apparatus) according to the manufacturer’s guidelines. Before mass spectrometry analysis, the evaporated peptide samples were dissolved in 2% ACN (v/v) with 0.05% aqueous trifluoroacetic acid (TFA) (v/v).

LC-MS/MS analysis was carried out using an Orbitrap FusionTM mass spectrometer (Thermo Fisher Scientific) with a New Objective digital PicoView 565 nanospray source (Scientific Instrument Services) coupled to a DionexTM UltiMateTM 3000 RSLC Nano Liquid chromatography. The peptides were loaded into an Acclaim PepMapTM 100 nano trap column (nanoViperTM C18, 0.3 × 5 mm, 5 µm particle size, 100 Å pore size; Thermo Fisher Scientific) with loading buffer (2% ACN with 0.05% aqueous TFA (v/v)) for 5 min desalting at a flow rate 5 μL/min. Next, the peptides were eluted onto an Acclaim PepMapTM RSLC C18 (nanoViperTM 75 μm × 25 cm, 2 µm particle size, 100 Å pore size, Thermo Fisher Scientific) kept at 50°C and separated by linear gradient elution over 64 min from 2%–25% B and 6 min gradient from 25%–60% B, followed by a 10 min wash step with 98% B, and 34 min of equilibration with 2% B. Mobile phase A was composed of LC-MS grade water and 0.1% formic acid (FA) while B was ACN 80% with 0.1% aqueous FA (v/v). The flow rate was 300 nL/min. The Orbitrap mass analyser was operated in positive ion mode, with the static positive ion spray voltage set to 2.45 kV, and ion transfer tube temperature to 275°C. The master scan was acquired at resolving power settings of 120,000 (FWHM @ m/z 200), precursor mass range 350–1,400 m/z. The MS/MS spectra of multiply charged ions were collected in data-dependent mode (top20 method of the most intensive precursors). Dynamic exclusion was set to 20 s. The peptides were fragmented using collision-induced dissociation (CID) with the normalized collision energy setting at 35%. The peptide fragments generated via CID were detected in an ion trap (rapid scan rate). The profile data was recorded in the master scan and the centroid data in MS/MS scans.

#### 4.2.1 Data processing

Data analysis was performed with the software Proteome Discoverer™ version 2.2 (Thermo Fisher Scientific). The database search was performed with the Sequest HT search engine against the cRap protein database (ftp://ftp.thegpm.org/fasta/cRAP) containing all p53 possible readthrough fasta sequences. The search engine settings employed 10 ppm precursor mass tolerance and 0.6 Da fragment mass tolerance, enzyme Trypsin (full), considering up to two missed cleavages and the following dynamic modifications: methionine oxidation (+15.995 Da); protein N-terminal acetylation (+42.011 Da), and static modification: cysteine carbamidomethylation (+57.021 Da). The results of the search were further submitted to generate the final report with the strict false discovery rate (FDR) threshold of 1% using a fixed value PSM validator. Using these results, extracted ion chromatograms (XICs) of selected peptides were analysed in Freestyle (Thermo Fisher scientific) software. The area under the XIC peak was then used to compare concentrations of different mutations within a sample (summarised in Figure 5). The mass spectrometry (MS) proteomics data have been deposited to the ProteomeXchange Consortium via the PRIDE partner repository with the dataset identifier PXD039307, Username: reviewer_pxd039307@ebi.ac.uk, Password: dPkP1Z4r.

The measured data was also subsequently reanalysed in PEAKS software (Figures 3, 4). The same database, and a combination of all possible p53 readthrough and common contaminants, was used. The search engine settings employed 10 ppm precursor mass tolerance and 0.6 Da fragment mass tolerance, enzyme Trypsin (full), considering up to two missed cleavages and the following dynamic modifications: methionine oxidation (+15.995 Da); protein N-terminal acetylation (+42.011 Da), and cysteine carbamidomethylation (+57.021 Da). The search was validated by a decoy database, aiming for 1% peptide FDR (Zhang et al., 2012). A secondary search was performed, looking for semi-tryptic peptides. The final result (Figures 3, 4) contains peptides from both. The PEAKS software automatically quantifies peptides abundance in a sample based on its extracted ion chromatogram in raw files. For this quantification, the area under the chromatographic peak of a peptides precursor ion is used, in essence it is a label free quantification without normalisation of samples. These peptide areas are listed in Figures 3, 4.

## Data Availability

The data presented in the study are deposited in the ProteomeXchange Consortium via the PRIDE partner repository, accession number: PXD039307; http://www.ebi.ac.uk/pride/archive/projects/PXD039307.
