# Supplementary material for: Misincorporations of amino acids in p53 in human cells at artificially constructed termination codons in the presence of the aminoglycoside Gentamicin
Source: Front Genet. 2024 Nov 5;15:1407375. doi: 10.3389/fgene.2024.1407375 (PMC11573534; doi:10.3389/fgene.2024.1407375)
Supplement: Supplementary file 1 [file DataSheet1.PDF]

Misincorporations of amino acids in p53 in human cells at artificially constructed termination stop codons in the presence of the aminoglycoside Gentamicin

Kamila Pawlicka[1^], Tomas Henek[2^], Lukas Uhrik[2^], Lenka Hernychova[2*], Monikaben Padariya[3], Jakub Faktor[3], Sławomir Makowiec[4], Borivoj Vojtesek[2,6], David Goodlett[5], Ted Hupp[1,3*], Umesh Kalathiya[3*]

[1]*University of Edinburgh, Institute of Genetics and Molecular Medicine, Edinburgh Cancer Research Centre, Edinburgh, Scotland, UK*
[2]*Research Centre for Applied Molecular Oncology, Masaryk Memorial Cancer Institute, Zluty kopec 7, 656 53 Brno, Czech Republic*
[3]*International Centre for Cancer Vaccine Science (ICCVS), University of Gdańsk, 80-309 Gdańsk, Poland*
[4]*Department of Organic Chemistry, Faculty of Chemistry, Gdańsk University of Technology, Narutowicza* Street 11/12, 80-233, Gdańsk, Poland
[5]Biochemistry & Microbiology, University of Victoria, Petch Bldg Room 207 Victoria BC, Canada
[6]Laboratory of Growth Regulators, Institute of Experimental Botany, The Czech Academy of Sciences , Olomouc, Czech Republic

^ *authors contributed equally to this work.*
*Corresponding author emails: lenka.hernychova@mou.cz, ted.hupp@ed.ac.uk, umesh.kalathiya@ug.edu.pl

Supporting Materials

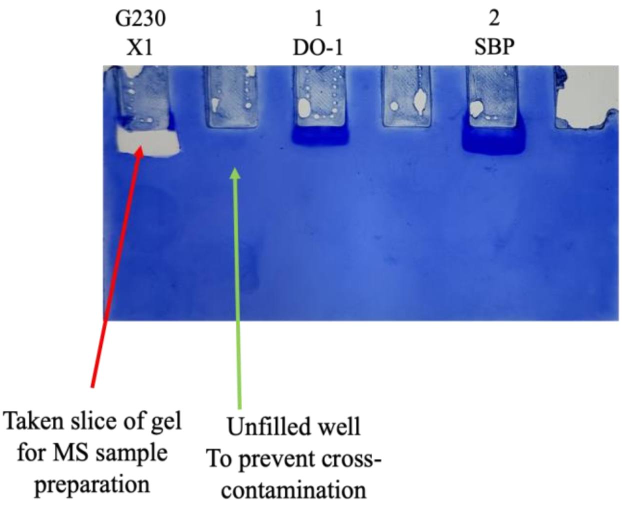

Supplementary Figure 1. Processing the p53 protein immunoprecipitate using an SDS polyacrylamide gel. Lysates from from immunoprecipitation with either the p53 antibody DO-1 (lane 1) or streptavidin beads (lane 2) were loaded onto an SDS-polyacrylamide gel and electrophoresis was carried out for 10 minutes just until the protein material could enter the gel. The proteins were stained with Coomassie brilliant blue G-250 and the gel containing proteins was cut (as in the lane X1) out and trypsinized. The red arrow highlights the region excised (lane G230 X1) as an example of the amount of gel excised for processing. The green arrow emphasizes that an empty lane separated each sample to minimize sample cross-over during electrophoresis.

A. Arg incoporation at the 213 stop codon position

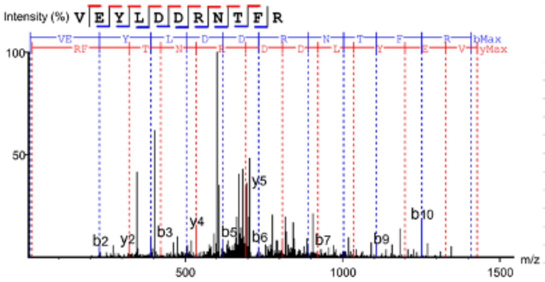

#	b	Seq	y	#
1	100.08	V		11
2	229.14	E	1328.82	10
3	392.23	Y	1199.84	9
4	505.28	L	1036.56	8
5	620.25	D	923.53	7
6	735.36	D	808.62	6
7	891.67	R	693.54	5
8	1005.42	N	537.34	4
9	1106.62	T	423.24	3
10	1253.71	F	322.25	2
11		R	175.12	1

B. A stable trytic peptide in the N-terminus of p53 that is the site of a new stop codon

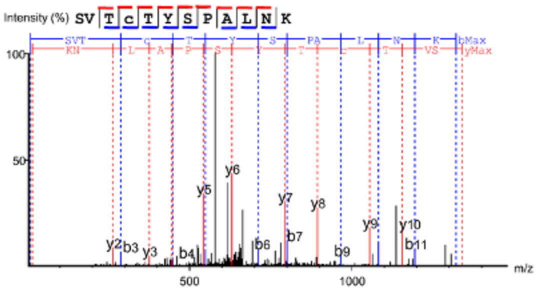

#	b	Seq	y	#
1	88.04	S		12
2	187.11	V	1253.62	11
3	288.23	T	1154.70	10
4	448.19	C(+57.02)	1053.70	9
5	549.32	T	893.59	8
6	712.29	Y	792.51	7
7	799.37	S	629.53	6
8	896.36	P	542.45	5
9	967.48	A	444.95	4
10	1080.67	L	374.26	3
11	1194.76	N	261.23	2
12		K	147.11	1

Supplementary Figure 2. Tryptic peptide coverage of the p53 protein expressed from the R213X expression plasmid. (A) An example of independent representative mass spectrometric run with tryptic peptides from DO-1 bead pull down, with the MS/MS spectrum for the 203-VEYLDDRNTFR-213 tryptic peptide and table with ion matches (red are *y*- ions and blue are *b*- ions). Additional parameters include; scan: 16 697; -10lgP PEAKS score: 47.67; mass of the precursor peak: 1426.63, m/z 714.35 (z = 2); error in ppm: 1.1; RT 27.10 min. This peptide was detected in Experiment 1; See Table 1A; column 3, row 21). (B) A stable tryptic peptide, 121-SVTCTYSPALNK-132, was detected frequently and this peptide was used to create a nested set of PTCs. Additional parameters include; scan: 14126; -10lgP PEAKS score: 74.57; mass of the precursor peak: 1339.64, m/z 670.83 (z = 2); error in ppm: 0.8; RT 24.78 min; see Figure 3*A*, columns 1-3, row 8.

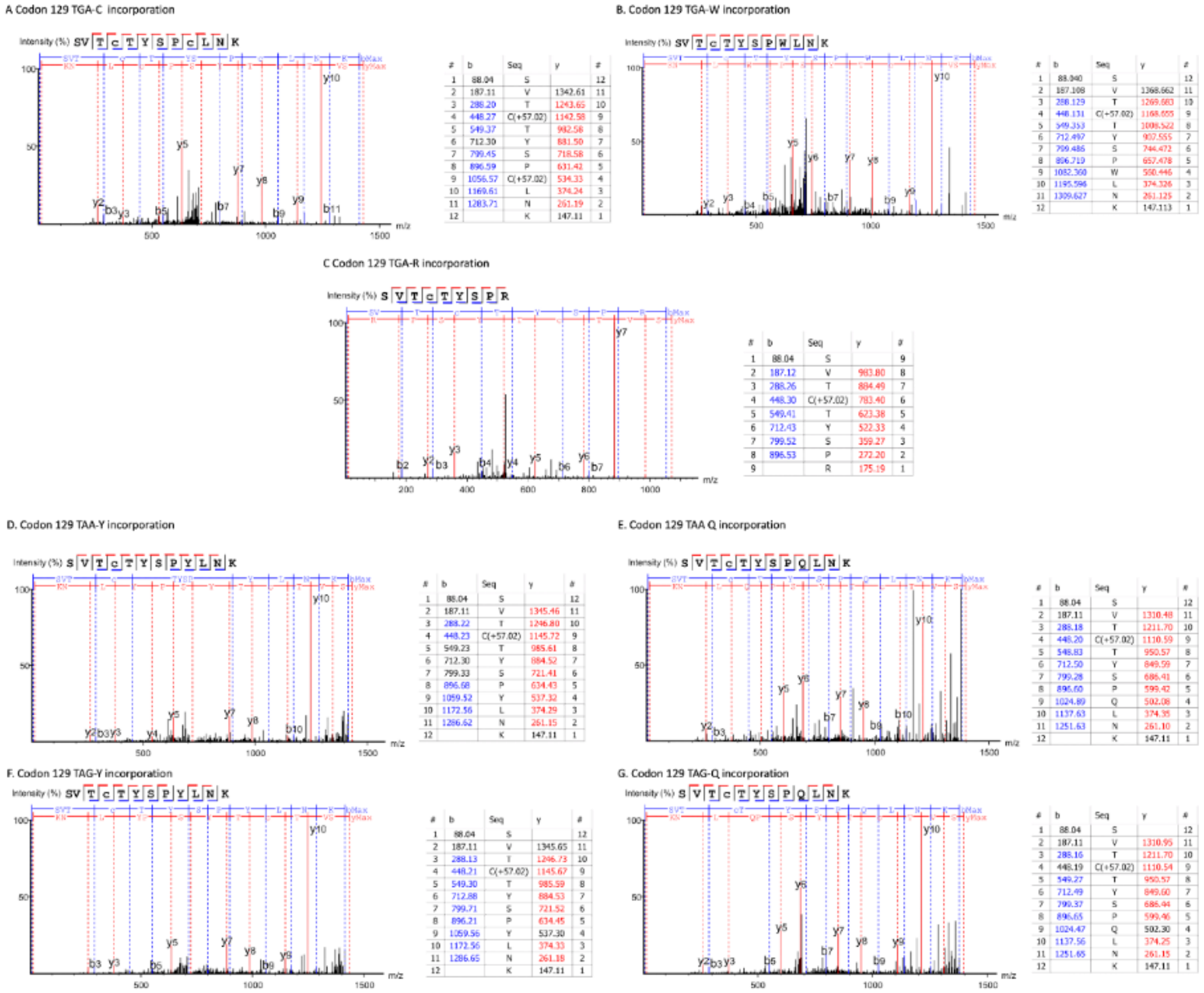

Supplementary Figure 3. Tryptic peptide coverage of the p53 protein expressed from different codon 129 premature termination codons. Representative spectra were collected as indicated: (A) TGA codon 129-C replacement (see Figure 4A, column 7, row 2e); (B) TGA codon 129-W replacement (see Figure 4*A*, column 7, row 2b); (C) TGA codon 129-R replacement (see Figure 4*A*, column 7, row 2a and Figure 4B, column 9, row 2a); (D). TAA codon 129-Y replacement (see Figure 4*B*, column 1, row 2d); (E). TAA codon 129-Q replacement (see Figure 4B, columns 1-3, row 2c); (F). TAG codon 129-Y replacement (see Figure 4B, columns 5 and 6, row 2d); and (G). TAG codon 129-Q replacement (see Table 2B, columns 4-6, row 2c). Further parameters from the measurement and evaluation are summarized: (A) scan: 14774; -10lgP PEAKS peptide score: 53.67; mass of the precursor peak: 1428.64, m/z 715.33 (z = 2); error in ppm: 0.4; RT 25.35 min; area: 3.37E5; (B) scan: 37293; -10lgP PEAKS peptide score: 47.94; mass of the precursor peak: 1454.94, m/z 728.35 (z = 2); error in ppm: -1.0; RT 47.43 min; area: nd.; (C) scan: 8474; -10lgP PEAKS peptide score: 55.77; mass of the precursor peak: 1069.49, m/z 535.75 (z = 2); error in ppm: 0.5; RT 18.93 min; area: 1.25E6; (D) scan: 27242; -10lgP PEAKS peptide score: 44.78; mass of the precursor peak: 1431.67, m/z 716.84 (z = 2); error in ppm: -0.1; RT 39.16 min; area: 2.13E5; (E) scan: 21770; -10lgP PEAKS peptide score: 48.37; mass of the precursor peak: 1396.67, m/z 699.34 (z = 2); error in ppm: 1.0; RT 32.73 min; area: 4.93E5; (F) scan: 28453; -10lgP PEAKS peptide score: 42.13; mass of the precursor peak: 1431.67, m/z 716.84 (z = 2); error in ppm: 1.9; RT 38.88 min; area: 4.93E5; (G) scan: 21717; -10lgP PEAKS peptide score: 61.41; mass of the precursor peak: 1396.67, m/z 699.34 (z = 2); error in ppm: 2.1; RT 37.88 min; area: 2.23E6.

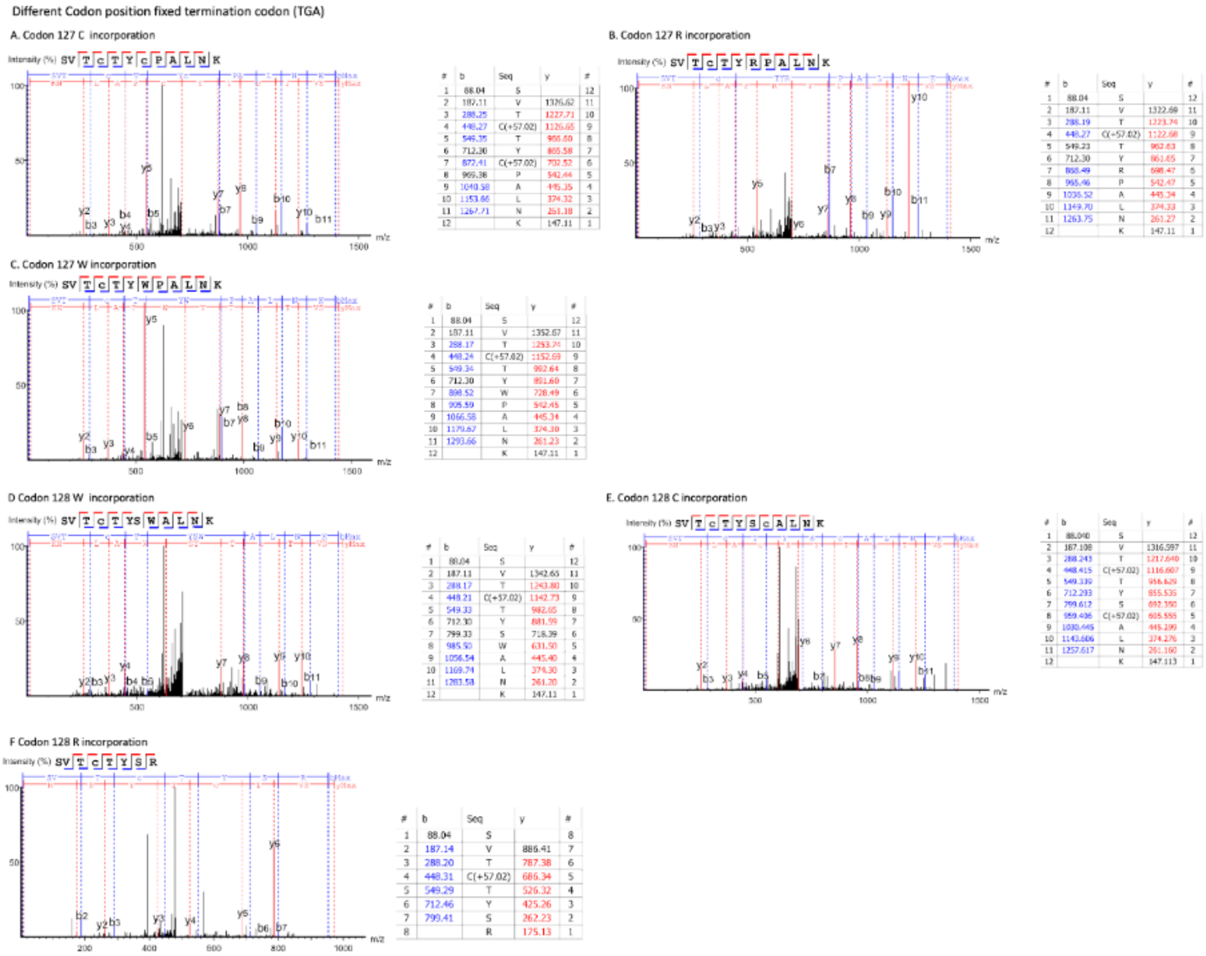

Supplementary Figure 4. Tryptic peptide coverage of the p53 protein expressed from different codon 127 and 128 premature termination codon TGA. Representative spectra were collected as indicated: (A) codon 127-C replacement (Figure 4A, column 9, row 4b and Figure 4B, column 13, row 4b); (B) codon 127-R replacement (Figure 4A, column 9, row 4d and Figure 4B, column 13, row 4d); (C) codon 127-W replacement (Figure 4A, column 9, row 4a and Figure 4B, column 13, row 4a); (D). codon 128-W replacement (Figure 4A, column 8, row 3a); (E) codon 128-C replacement (Figure 4A, column 8, row 3c and Figure 4B, column 10, row 3c); and (F) codon 128-R replacement (Figure 4A, column 8, row 3b). Further are introduced parameters from the measurement and evaluation: (A) scan: 16092; -10lgP PEAKS peptide score: 51.14; mass of the precursor peak: 1412.64, m/z 707.32 (z = 2); error in ppm: -0.6; RT 26.11 min; area: 2.34E6; (B) scan: 10595; -10lgP PEAKS peptide score: 56.64; mass of the precursor peak: 1408.71, m/z 705.36 (z = 2); error in ppm: -1.8; RT 20.81 min; area: 1.41E6; (C) scan: 26746; -10lgP PEAKS peptide score: 69.94; mass of the precursor peak: 1438.69, m/z 720.35 (z = 2); error in ppm: 0.7; RT 36.49 min; area: 3.16E6; (D) scan: 22501; -10lgP PEAKS peptide score: 31.14; mass of the precursor peak: 1428.67, m/z 715.34 (z = 2); error in ppm: 0.7; RT 33.71 min; area: 1.11E5; (E) scan: 24451; -10lgP PEAKS peptide score: 48.05; mass of the precursor peak: 1412.64, m/z 707.33 (z = 2); error in ppm: 2.2; RT 35.21 min; area: 8.94E5; (F) scan: 5880; -10lgP PEAKS peptide score: 39.54; mass of the precursor peak: 972.43, m/z 487.22 (z = 2); error in ppm: -0.4; RT 16.31 min; area: 1.25E5.

A. Samples 1-3. TAA STOP codon 129; Q>Y.

Sample			TAA YSP*L								
			S1			S2			S3		
Sequence	Charge	m/z of precursor ion	RT	Area MI	Ratio	RT	Area MI	Ratio	RT	Area MI	Ratio
[K].SVTCTYSP**Y**LNK.[M]	2	716.8425	39.1424	2,387,035	**37%**	38.6771	1,628,360	**31%**	38.8245	1,301,315	**17%**
[K].SVTCTYSP**Q**LNK.[M]	2	699.3401	33.0285	4,037,734	**63%**	32.7167	3,680,403	**69%**	32.7742	6,182,903	**83%**

B. Samples 4-6. TAG STOP codon 129; Q>Y.

Sample			TAG YSP*L								
			S4			S5			S6		
Sequence	Charge	m/z of precursor ion	RT	Area MI	Ratio	RT	Area MI	Ratio	RT	Area MI	Ratio
[K].SVTCTYSP**Y**LNK.[M]	2	716.8425	38.7917	6,194,752	**26%**	38.8221	4,648,152	**21%**	38.8328	8,152,441	**22%**
[K].SVTCTYSP**Q**LNK.[M]	2	699.3401	32.7050	17,524,788	**74%**	32.7222	17,473,129	**79%**	32.7309	29,728,200	**78%**

C. Samples 7-9. TGA STOP codon 129; R>W.

			TGA YSP*L								
Sample			S7			S8			S9		
Sequence	Charge	m/z of precursor ion	RT	Area MI	Ratio	RT	Area MI	Ratio	RT	Area MI	Ratio
[K].SVTCTYSP**W**LNK.[M]	2	728.3505	47.4298	1,678,544	**30%**	47.45**	848,058	**23%**	47.4801	1,512,763	**19%**
[K].SVTCTYSP**R**.[L]	2	535.7504	22.87*	3,894,371	**70%**	22.88**	2,820,608	**77%**	22.9839	6,596,849	**81%**

* not identified, based on MS1 only
** not identified, based on MS1 only, lower quality

D. Samples 10-12. TGA STOP codon 128

			TGA YS*AL								
			S7			S8			S9		
			RT	Area MI	Ratio	RT	Area MI	Ratio	RT	Area MI	Ratio
[K].SVTCTYS**C**ALNK.[M]	2	702.3183	31.5416	3,660,841	-	ND	-	-	ND	-	-

E. Samples 13-15. TGA STOP codon 127

			TGA Y*SPAL								
Sample			S13			S14			S15		
Sequence	Charge	m/z of precursor ion	RT	Area MI	Ratio	RT	Area MI	Ratio	RT	Area MI	Ratio
[K].SVTCTY**R**PALNK.[M]	2	705.3639	26.4834	5,473,438	**14%**	26.44**	166,079	**16%**	ND	-	-
[K].SVTCTY**W**PALNK.[M]	2	720.3530	51.9013	22,109,575	**58%**	52.02**	876,743	**84%**	ND	-	-
[K].SVTCTY**C**PALNK.[M]	2	707.3287	35.1758	10,793,744	**28%**	ND	-	-	ND	-	-
[K].SVTCTY**R**PALNK.[M]	*3*	*470.5784*	*26.5*	*20,327,641*		*26.4*	*1,671,036*				
				R	***44%***		*R*	***68%***			
				W	***38%***		*W*	***32%***			
				C	***18%***						

** not identified, only based on MS1, lower quality

Supplementary Figure 5. Summary of % amino acid incorporation in two independent experiments quantified using Proteome Discoverer™ version 2.2 and used to create the *Figure 5* summary. The

indicated samples are: (A). TAA codon 129, in triplicates, highlighting more Q substitutions (63%, 69%, and 83%) over Y substitutions (37%, 31%, and 17%), with cross reference to Figure 4B, columns 1-3, rows 2C and 2D; (B). TAG codon 129, in triplicates, highlighting more Q substitutions (74%, 79%, and 78%) over Y substitutions (26%, 21%, and 22%), with cross reference to Figure 4*B*, columns 4-6, rows 2C and 2D; (C). TGA codon 129, in triplicates, highlighting more R substitutions (70%, 77%, and 81%) over W substitutions (30%, 23%, and 19%), with cross reference to Figure 4*B*, columns 7-9, rows 2A and 2B; (D). TGA codon 128, in triplicates, highlighting mass of C substitutions, with cross reference to Figure 4*B*, columns 10-12, row 2E, which was not detected using PEAKS; (E). TGA codon 127, in triplicates, highlighting distribution (in %) or R, W, and C (charge +2 state), as well R residue in the charge +3 state, with cross reference to Figure 4*B*, columns 13-15, rows 4 A-D. Although we detect both 2+ charge and 3+ charge peptide abundance in codon 127 using the Proteome Discoverer™ version 2.2, Peaks only detects the 2+ charge results. Thus, for this codon (see Figure 5, 'W" dominates over R).
